# Dietary Probiotic *Pediococcus acidilactici* GKA4, Dead Probiotic GKA4, and Postbiotic GKA4 Improves Cisplatin-Induced AKI by Autophagy and Endoplasmic Reticulum Stress and Organic Ion Transporters

**DOI:** 10.3390/nu16203532

**Published:** 2024-10-18

**Authors:** Jaung-Geng Lin, Wen-Ping Jiang, You-Shan Tsai, Shih-Wei Lin, Yen-Lien Chen, Chin-Chu Chen, Guan-Jhong Huang

**Affiliations:** 1 School of Chinese Medicine, College of Chinese Medicine, China Medical University, Taichung 404, Taiwan; jglin@mail.cmu.edu.tw; 2 Chinese Medicine Research Center, China Medical University, Taichung 404, Taiwan; 3 School of Pharmacy, College of Pharmacy, China Medical University, Taichung 404, Taiwan; wpjiang@mail.cmu.edu.tw; 4 Biotech Research Institute, Grape King Bio Ltd., Taoyuan City 325, Taiwanlan.chen@grapeking.com.tw (Y.-L.C.); 5 Institute of Food Science and Technology, National Taiwan University, Taipei 106, Taiwan; gkbioeng@grapeking.com.tw; 6 Department of Food Sciences, Nutrition, and Nutraceutical Biotechnology, Shih Chien University, Taipei 104, Taiwan; 7 Department of Bioscience Technology, Chung Yuan Christian University, Taoyuan 320, Taiwan; 8 Department of Food Nutrition and Healthy Biotechnology, Asia University, Taichung 413, Taiwan; 9 Department of Chinese Pharmaceutical Sciences and Chinese Medicine Resources, College of Chinese Medicine, China Medical University, Taichung 404, Taiwan

**Keywords:** *Pediococcus acidilactici* GKA4, cisplatin, acute kidney injury, oxidative stress, apoptosis, autophagy, ER stress, transporter proteins

## Abstract

Background/Objectives: Acute kidney injury (AKI) syndrome is distinguished by a quick decline in renal excretory capacity and usually diagnosed by the presence of elevated nitrogen metabolism end products and/or diminished urine output. AKI frequently occurs in hospital patients, and there are no existing specific treatments available to diminish its occurrence or expedite recovery. For an extended period in the food industry, *Pediococcus acidilactici* has been distinguished by its robust bacteriocin production, effectively inhibiting pathogen growth during fermentation and storage. Methods: In this study, the aim is to assess the effectiveness of *P. acidilactici* GKA4, dead probiotic GKA4, and postbiotic GKA4 against cisplatin-induced AKI in an animal model. The experimental protocol involves a ten-day oral administration of GKA4, dead probiotic GKA4, and postbiotic GKA4 to mice, with a cisplatin intraperitoneal injection being given on the seventh day to induce AKI. Results: The findings indicated the significant alleviation of the renal histopathological changes and serum biomarkers of GKA4, dead probiotic GKA4, and postbiotic GKA4 in cisplatin-induced nephrotoxicity. GKA4, dead probiotic GKA4, and postbiotic GKA4 elevated the expression levels of HO-1 and decreased the expression levels of Nrf-2 proteins. In addition, the administration of GKA4, dead probiotic GKA4, and postbiotic GKA4 significantly reduced the expression of apoptosis-related proteins (Bax, Bcl-2, and caspase 3), autophagy-related proteins (LC3B, p62, and Beclin1), and endoplasmic reticulum (ER) stress-related proteins (GRP78, PERK, ATF-6, IRE1, CHOP, and Caspase 12) in kidney tissues. Notably, GKA4, dead probiotic GKA4, and postbiotic GKA4 also upregulated the levels of proteins related to organic anion transporters and organic cation transporters. Conclusions: Overall, the potential therapeutic benefits of GKA4, dead probiotic GKA4, and postbiotic GKA4 are significant, particularly after cisplatin treatment. This is achieved by modulating apoptosis, autophagy, ER stress, and transporter proteins to alleviate oxidative stress.

## 1. Introduction

Cisplatin (cis-diamminedichloroplatinum II) is employed for treating a range of cancers, such as bladder, ovarian, lung, and testicular cancer [1]. Its efficacy in impeding cancer growth has led to its application in 10–20% of cancer patients. Nonetheless, the reabsorption of cisplatin or its metabolites by local transporters in the proximal tubules leads to the targeted destruction of malignant tumors and severe harm to proximal tubular cells. This, in turn, contributes to complications like peripheral neuropathy, allergies, and acute kidney injury (AKI) [2]. The regulatory pathways underlying cisplatin-induced acute kidney injury (AKI) are not fully understood, and several studies have demonstrated the role of renal reactive oxygen species (ROS) in AKI. By accumulating in the kidney, cisplatin induces the production of free radicals in cells, impairing renal tubular cells. Hence, the antioxidant defense system is a critical marker for cisplatin-induced nephrotoxicity [3].

Oxidative stress serves as the foundational mechanism for AKI, playing a critical role in the chain reaction of ROS and metabolites activating receptors. Nuclear factor erythroid 2-related factor (Nrf2) is a primary transcription factor that oversees cellular oxidative stress and serves as a regulator for maintaining intracellular redox stability by controlling antioxidant proteins [4]. Research findings indicate the importance of Nrf2 in influencing endogenous antioxidant genes and redox systems, playing a critical role in protecting against cytoprotective responses and apoptotic lesions [5,6].

Excessive nephrotoxic doses of cisplatin may lead to a notable elevation in kidney necrosis and apoptosis [7], and cisplatin has been confirmed to activate the apoptotic pathway in mitochondria [8]. When renal epithelial cells are treated with cisplatin, there is a translocation of Bax to mitochondria and a subsequent activation of caspases [9]. Moreover, caspase, which is identified as a cell death protease, assumes a critical role in the apoptotic execution phase of cisplatin-induced renal tubular epithelial cell death both in vitro and in vivo. Caspase 3 was activated within 12 h of cisplatin treatment in renal epithelial cells, and inhibiting caspase activity significantly mitigated cisplatin-induced cell death [10]. Additionally, autophagy acts as a bulk protein degradation system, potentially playing a pivotal role in maintaining renal homeostasis [11]. This intricate process is primarily governed by proteins encoded by autophagy-related genes (Atg), including Atg5, Atg6 (beclin1), Atg7, and LC3 (a mammalian homolog of yeast Atg8) [12]. Experiments have shown that autophagy dysregulation is involved in the pathophysiology of AKI. For example, the knockdown of Atg7 in proximal renal tubular cells inhibits autophagy, causing loss of renal function, increased tissue damage, and more tubular apoptosis in ischemic AKI [13].

Endoplasmic reticulum (ER) stress stands out as a pivotal pathogenic mediator in a multitude of renal diseases, and the prospect of inhibiting ER stress has emerged as a potential therapeutic avenue for affected patients [14]. Various stimuli, including hypoxia, metabolic dysfunction, and the accumulation of mutant proteins, can incite ER stress in the context of AKI. The stress response is triggered by the accumulation of misfolded proteins in the ER lumen, initiating the unfolded protein response (UPR). The UPR encompasses three main pathways: the PERK-eIF2α-ATF4 pathway, the IRE1-XBP1 pathway, and the ATF6 pathway [15]. In the dormant state of the UPR, the molecular chaperone GRP78 binds to PERK, ATF6, and IRE1, preventing their activation and signaling. However, in the presence of accumulated misfolded proteins, the dissociation of GRP78 from these sensors allows for downstream phosphorylation and signaling [8].

Xenobiotics and endogenous by-products are secreted and reabsorbed primarily in proximal tubule cells [16]. The movement of chemicals into the kidneys across basolateral membranes is facilitated by secondary active transport systems such as organic cation transporters (OCTs) and organic anion transporters (OATs). OATs are structurally similar to OCTs and belong to the *SLC22A* gene family [17]. Experimental nephrotoxicity is characterized by a significant reduction in tubular cation clearance, as indicated in previous studies [18]. The decline in tubular cation clearance observed during experimental nephrotoxicity results from the downregulation of tubular organic anion and cation transporter expression [19]. Specific clearance mechanisms have evolved in biological systems to counteract the adverse effects of metabolites, with OCTs and OATs serving pivotal roles in the renal clearance of toxins, xenobiotics, and exogenous substrates, including frequently used medications [20].

*P. acidilactici*, a Gram-positive cocci and facultative anaerobic lactobacilli, commonly presents in pairs or quadruples, displaying adaptability to the diverse growth conditions that facilitate its colonization of the digestive tract [21]. In contrast, *P. acidilactici*, recognized for its bacteriocin production, possesses the ability to colonize the human intestine. It imparts positive effects, including the adjustment of intestinal bacteria composition, reconstruction of the intestinal mucosal barrier function, and enhancement of the defense mechanisms of the digestive tract [22,23]. Despite these acknowledged benefits, there is a paucity of reports on probiotic products demonstrating renoprotective effects. In this study, we explore the in vivo therapeutic applications of orally administered GKA4, dead probiotic GKA4, and postbiotic GKA4 [24,25]. The findings suggest that GKA4, dead probiotic GKA4, and postbiotic GKA4 hold promise as a probiotic candidate for mitigating cisplatin-induced AKI, indicating its potential as a supplementary food for AKI prevention.

## 2. Materials and Methods

### 2.1. Preparation of Samples

*P. acidilactici* GKA4 was isolated from pickles and preserved at BCRC, Taiwan, with No. 910876. Cultivation of strain GKA4 occurred in a 10-ton fermenter at 37 °C for 16 h, utilizing an 80% medium solution consisting of 5% glucose, 2.0% yeast extract, 0.05% MgSO_4_, 0.1% K_2_HPO_4_, and 0.1% Tween 80 while maintaining a pH of 6.0. After incubation, the fermented GKA4 was centrifuged. The pellet was mixed with protectant and freeze-dried at 25 °C for 48 h. The live bacteria powder was applied to the animal model. For the preparation of dead probiotic GKA4, the live GKA4 pellet was suspended with water and then autoclaved under 121 °C for 15 min. The autoclaved GKA4 was freeze-dried at 25 °C for 48 h. For the preparation of postbiotic GKA4 powder, the fermented supernatant was collected. After fermentation, the pH of the supernatant was modified to 6.8–7.2, followed by heating at 121 °C for 3 min. Then, the supernatant was freeze-dried at 25 °C for 48 h.

### 2.2. Reagents

Cisplatin, *N*-acetyl cysteine (NAC), and additional reagents were obtained from Sigma–Aldrich (St. Louis, MO, USA) for the study. Detection kits for creatinine (CRE) and blood urea nitrogen (BUN) were obtained from HUMAN Diagnostics Worldwide (Wiesbaden, Germany). The antibodies used for Beclin 1 (1:1000), LC3B (1:1000), P62 (1:1000), Bax (1:1000), Bcl-2 (1:1000), and caspase-3 (1:1000) were from Cell Signaling Technology (Beverly, MA, USA). Antibodies against GRP87 (1:1000), PERK (1:1500), ATF-6 (1:1500), IRE1 (1:1000), CHOP (1:1500), caspase 12 (1:1000), heme oxygenase-1 (HO-1) (1:2000), Nrf2 (1:1500), and and β-actin (1:10000) were supplied by Abcam (Cambridge, UK). The loading control utilized in this experiment is β-actin.

### 2.3. Animals

Eight-week-old ICR male mice (32 ± 3 g) from BioLASCO Taiwan Co., Ltd. (Yi-Lan Breeding Center, Yi-Lan County, Taiwan) were used as experimental subjects. Before commencing the experiment, the mice were acclimated for 3–5 days in a 12 h light/dark cycle environment maintained at 23 °C and 50% relative humidity. The Animal Protection Committee of China Medical University approved the entire experimental protocol before the commencement of the experiment (CMUIACUC-2022-039).

### 2.4. Research Design

The experiment involved thirty-six male ICR mice which were randomly assigned to six groups (*n* = 6): (1) control, treated with saline (i.p.); (2) cisplatin, receiving cisplatin (20 mg/kg, i.p.); (3) NAC (300 mg/kg, i.p.) + cisplatin (20 mg/kg, i.p.); (4) GKA4 (250 mg/kg, oral suspension) + cisplatin (20 mg/kg, i.p.); (5) dead probiotic GKA4 (250 mg/kg, oral suspension) + cisplatin (20 mg/kg, i.p.); and (6) GKA4 postbiotics (250 mg/kg, oral suspension) + cisplatin (20 mg/kg, i.p.).

**Figure 1:**
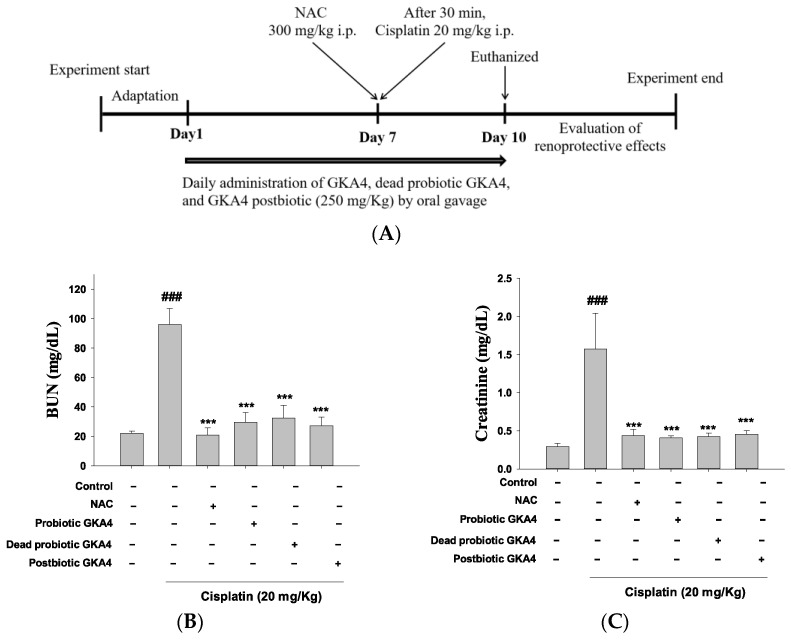

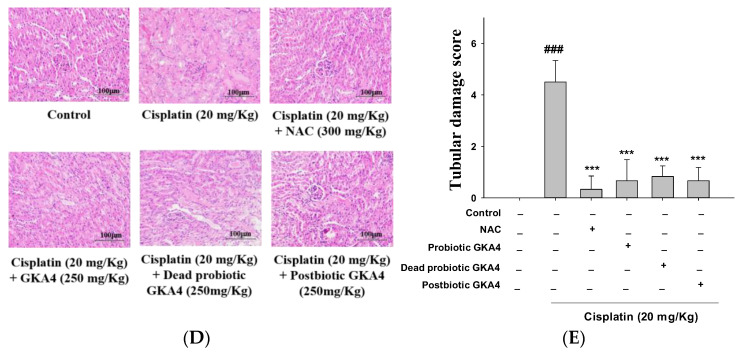
The experimental framework (**A**) and the renoprotective efficacy of GKA4, dead probiotic GKA4, and postbiotic GKA4 alleviates neurotoxicity induced by cisplatin treatment. Oral administration of GKA4, dead probiotic GKA4, and postbiotic GKA4 at 250 mg/kg was carried out daily for 10 consecutive days, with cisplatin being administered one hour after the seventh dose. The sacrifice of the mice occurred on the eleventh day. The levels of BUN (**B**) and CRE in the serum (**C**), the renal sections stained with H&E (400×) (**D**), and the renal injury scale (**E**) were assessed. Means ± S.E.M (*n* = 5) are shown in the presentation of the data. Statistical significance (*p* < 0.001) is indicated by ^###^ when compared with the control group sample. Statistical significance at *** *p* < 0.001 was evident in contrast to the cisplatin group.

Over 10 days, mice were orally gavaged with GKA4, dead probiotic GKA4, or GKA4 postbiotic at a dose of 250 mg/kg, administered once daily. The treatment protocol was initiated 7 days before cisplatin injection and persisted for 3 days following the injection. Saline was administered orally to the control group. The induction of AKI occurred on day 7 with an intraperitoneal injection of cisplatin (20 mg/kg) in the cisplatin, GKA4, dead probiotic GKA4, and GKA4 postbiotic groups. Blood samples were collected 72 h after cisplatin injection, while mice were anesthetized and stored at −20 °C for serum biomarker analysis before their kidneys were harvested at sacrifice 72 h post-cisplatin injection. Additionally, during this period, weekly body weight measurements were taken and averaged (Figure 1A) [26].

### 2.5. Kidney Index

Body weight assessments were conducted on mice before their sacrifice. Following the sacrifice, the kidneys were isolated and weighed. The kidney index was then determined by calculating the ratio of kidney weight (mg) to body weight (g) using the specified formula.

### 2.6. Renal Biomarker Measurements

The BUN and CRE levels were analyzed using an ELISA reader, serving as indicators for the evaluation of renal function (Roche Diagnostics, Cobas Mira Plus, Mannheim, Germany).

### 2.7. Histological Examination

Sections of kidney tissue (5 µm) were subjected to H&E processing and subsequently captured through light microscopy (Nikon, Eclipse, TS100, Japan). The severity of renal injury was evaluated and graded into five categories (normal, <25% light damage, 25–50% moderate damage, 50–75% heavy damage, and >75% severe damage), with numerical ratings being assigned from 0 to 4 [26].

### 2.8. The TBARS (Thiobarbituric Acid Reactive Substance) Assay

Measurement of malondialdehyde (MDA) levels served as the basis for detecting thiobarbituric acid reactive substances (TBARS), indicative of renal lipid peroxidation [27]. Lysis of the kidneys was performed using an ice-cold lysis buffer. Extracts were then combined with a thiobarbituric acid (TBA) solution at 90 °C for 45 min, generating the malondialdehyde (MDA)-TBA adduct. TBARS formation was quantified at 532 nm about a blank.

### 2.9. Glutathione (GSH) Assay

The GSH assay was carried out by reacting with DTNB (5, 5′-dithiobis (2-nitrobenzoic acid)). In a nutshell, a mixture of 100 μL of supernatant, 200 μL of 0.3 M phosphate buffer (pH 8.4), 400 μL of double-distilled water, and 500 μL of Ellman’s reagent was prepared, and a spectrophotometric analysis was conducted to measure absorbance at 412 nm [28]. The total protein concentration was determined using a dye-binding method, which adheres to the principles of the Bradford assay (Bio-Rad Laboratories, Hemel Hempstead, UK).

### 2.10. Western Blot Analysis

The kidney tissue lysate was prepared by homogenization in cold RIPA buffer containing protease inhibitors. The primary antibodies were employed for subsequent electrophoresis. Anti-rabbit or anti-mouse IgG antibodies conjugated to HRP were used for secondary antibody detection. HRP-labeled membrane-bound protein bands were visualized with chemiluminescent reagents, and the signals were recorded using Kodak Molecular Imaging Software 4.0 (Eastman Kodak Company, Rochester, NY, USA).

### 2.11. Statistical Analysis

Experimental results are displayed with the mean ± standard error of the mean (S.E.M). After confirming normal data distribution, a one-way ANOVA (analysis of variance) was executed, employing a Scheffé test for post hoc analysis, with the statistical significance being defined at *p* < 0.05.

## 3. Results

### 3.1. GKA4, Dead Probiotic GKA4, and Postbiotic GKA4 Exhibit Inhibitory Properties against Kidney Failure Leading to Improved Kidney Function Following Cisplatin-Induced AKI

The experimental design is depicted in Figure 1A. BUN and CRE serve as critical markers for assessing kidney function. Demonstrated in Figure 1B,C is the inhibitory effect on CRE and BUN levels following cisplatin-induced AKI through the oral administration of GKA4, dead probiotic GKA4, and postbiotic GKA4 (250 mg/kg). In current cancer treatment practices, *N*-acetylcysteine (NAC) is used to alleviate adverse effects and boost the efficacy of anticancer medications. Therefore, the outcomes indicate that pretreatment with GKA4, dead probiotic GKA4, and postbiotic GKA4 contributes to improved kidney function during cisplatin-induced AKI.

Subsequently, a renal tissue histopathology analysis was employed to assess the potential ameliorative effects of GKA4, dead probiotic GKA4, and postbiotic GKA4 on cisplatin-induced AKI. In Figure 1D, it can be observed that the control group had structurally normal kidney tissue. Conversely, the renal tissue subjected to cisplatin-induced AKI exhibited damage marked by the infiltration of inflammatory cells, vacuolar degeneration, tubular dysfunction, and necrosis. Consequently, GKA4, dead probiotic GKA4, and postbiotic GKA4, when orally administered, diminish kidney damage in mice, as evidenced by histological changes depicted in Figure 1D,E. Moreover, the orally administering GKA4, dead probiotic GKA4, and postbiotic GKA4 significantly lowered renal failure scores compared to cisplatin-induced nephrotoxicity, as depicted in Figure 1E. Thus, GKA4, dead probiotic GKA4, and postbiotic GKA4 play a role in improving renal activity in cisplatin-induced AKI.

### 3.2. Alterations in the Renal Index Were Examined in Mice Subjected to Cisplatin Treatment and Concomitant Administration of GKA4, Dead Probiotic GKA4, and Postbiotic GKA4

As depicted in Table 1, the cisplatin-treated mice exhibited a reduction in body weight and an elevation in the kidney index. Moreover, it was observed that the oral intake of GKA4, dead probiotic GKA4, and postbiotic GKA4 supplementation was associated with a notable improvement in resistance to attenuate nephrotoxicity induced by cisplatin along with a decrease in the renal index.

### 3.3. GKA4, Dead Probiotic GKA4, and Postbiotic GKA4 Alleviate Oxidative Stress in Cisplatin-Challenged AKI

**Figure 2:**
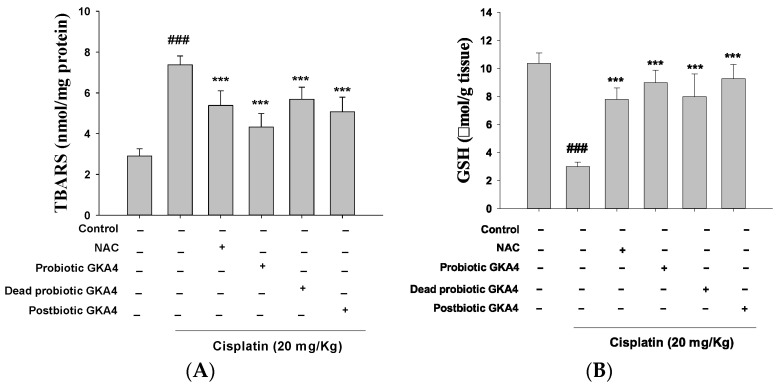
GKA4, dead probiotic GKA4, and postbiotic GKA4 alleviate oxidative stress in cisplatin-challenged AKI. The levels of MDA (**A**) and GSH (**B**) were assessed through specific assays for MDA and GSH. Means ± S.E.M (*n* = 5) are shown in the presentation of the data. Statistical significance (*p* < 0.001) is indicated by ^###^ when compared with the control group sample. *** *p* < 0.001 compared with the cisplatin group.

Reactive oxygen species (ROS) are essential initiators of apoptosis under different physiological and pathophysiological conditions, with particular importance being attributed to oxidative stress. As illustrated in Figure 2A,B, when compared to the control group, cisplatin treatment caused a reduction in GSH levels, indicating decreased antioxidant capacity and an elevation in MDA levels, reflecting increased lipid oxidation. Furthermore, oral administration of GKA4, dead probiotic GKA4, and postbiotic GKA4 resulted in decreased MDA levels and increased GSH content. Thus, GKA4, dead probiotic GKA4, and postbiotic GKA4 can reduce oxidative stress in AKI induced by cisplatin.

### 3.4. The Administration of GKA4, Dead Probiotic GKA4, and Postbiotic GKA4 in Cisplatin-Induced Nephrotoxicity Enhances Renal Antioxidant Defense and Promotes Activation of the HO-1/Nrf2 Signaling Pathway

**Figure 3:**
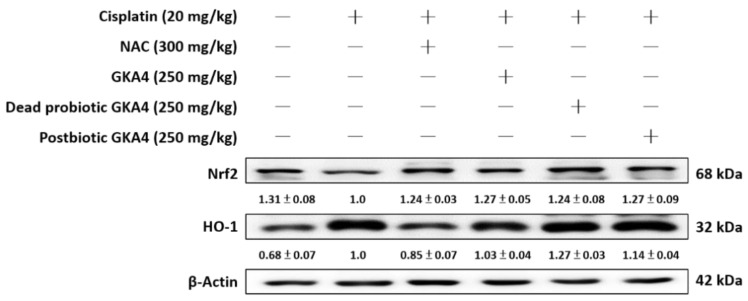
The effects of GKA4, dead probiotic GKA4, and postbiotic GKA4 on cisplatin-induced protein expression, including that of HO-1 and Nrf2, were investigated in kidney tissues. The expression of HO-1 and Nrf2 proteins in renal homogenates was assessed via Western blot analysis after exposure to cisplatin. Densitometric analysis was employed to assess the protein bands. The experiments were conducted independently at least three times and representative images were presented.

The presence of cisplatin within tubular cells results in the kidney facing significant susceptibility to its toxic impacts, including the induction of ROS production. Such excessive ROS activity attacks lipids, DNA, and other vital cellular macromolecules, leading to cellular demise and AKI [22]. Figure 3 demonstrates that the cisplatin group displayed lower basal levels of Kelch-like ECH-associated protein 1 (Keap1) expression and elevated levels of HO-1 expression compared to the control group. The administration of GKA4, dead probiotic GKA4, and postbiotic GKA4 via the oral route is capable of improving the expression levels of Nrf2 and HO-1 in comparison to the effects observed in the cisplatin group (Figure 3). The study revealed that GKA4, dead probiotic GKA4, and postbiotic GKA4 have the potential to enhance the expression of antioxidant enzyme-related proteins in the presence of cisplatin treatment.

### 3.5. GKA4, Dead Probiotic GKA4, and Postbiotic GKA4 Mitigate the Apoptosis Signaling Pathway Induced by Cisplatin

**Figure 4:**
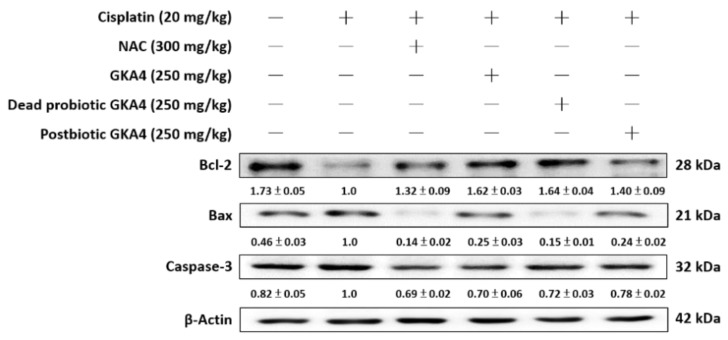
GKA4, dead probiotic GKA4, and postbiotic GKA4 administration resulted in changes in the expression levels of Bax, Bcl-2, and caspase-3 proteins after exposure to cisplatin. Antibodies specific to Bax, Bcl-2, caspase-3, and β-actin were used to conduct a Western blot analysis on kidney tissue lysates. A densitometric analysis was employed to assess the protein bands. The experiments were conducted independently at least three times, and representative images were presented.

For further elucidation of the anti-apoptotic effect of GKA4, dead probiotic GKA4, and postbiotic GKA4 after cisplatin exposure, immunoblotting was employed to analyze the expression levels of apoptosis-related proteins, including Bax, Bcl-2, and caspase-3. In comparison to the control group, the cisplatin group demonstrated increased protein expressions for Bax and caspase-3 and decreased expressions for Bcl-2. Conversely, pretreatment with GKA4, dead probiotic GKA4, and postbiotic GKA4 administration led to a marked decrease in Bax and caspase-3 levels while showing an increase in Bcl-2 levels following cisplatin exposure (Figure 4).

### 3.6. GKA4, Dead Probiotic GKA4, and Postbiotic GKA4 Mitigate the Autophagy Signaling Pathway Induced by Cisplatin

**Figure 5:**
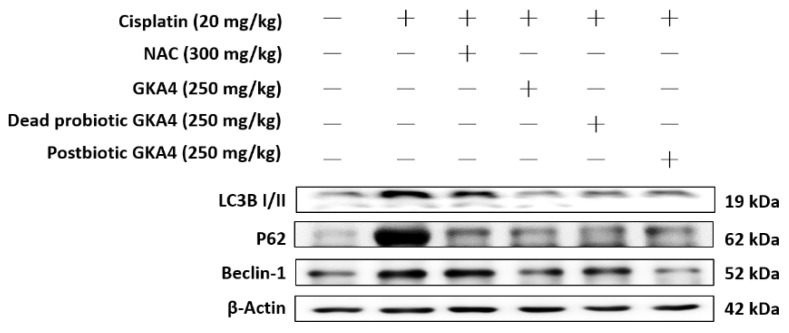
GKA4, dead probiotic GKA4, and postbiotic GKA4 resulted in a reduction in the levels of LC3B, P62, and Beclin 1 protein following exposure to cisplatin. Antibodies specific to LC3B, P62, Beclin 1, and β-actin were employed for a Western blot analysis of kidney tissue lysates. Protein bands were analyzed via densitometric analysis. The experiments were replicated at least three times, and representative images were displayed.

Autophagy is essential for maintaining cellular homeostasis. In comparison to the control group, the cisplatin group showed elevated protein expressions of LC3B, P62, and Beclin 1. In contrast, the levels of LC3B, P62, and Beclin 1 were markedly suppressed by GKA4, dead probiotic GKA4, and postbiotic GKA4 after exposure to cisplatin (Figure 5).

### 3.7. GKA4, Dead Probiotic GKA4, and Postbiotic GKA4 Alleviates the Cisplatin-Induced ER Stress Expressions

**Figure 6:**
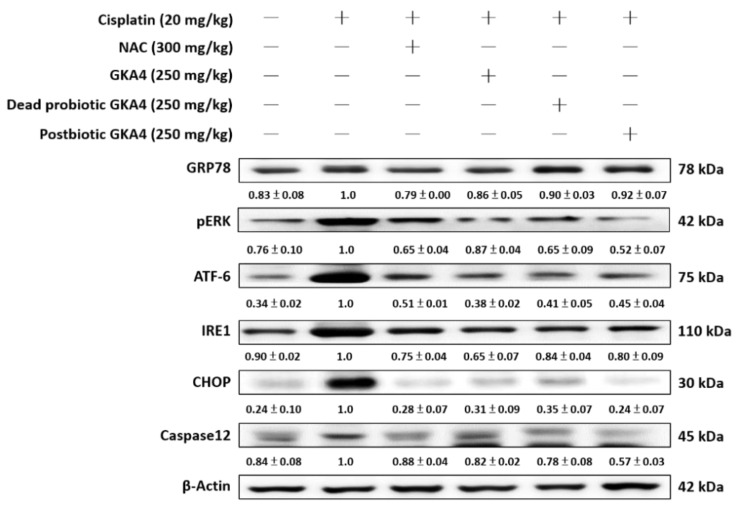
GKA4, dead probiotic GKA4, and postbiotic GKA4 modulated the expression of ER stress proteins in mice with cisplatin-induced AKI. A Western blot analysis was performed on kidney tissue lysates to evaluate protein expression using antibodies specific to GRP78, PERK, ATF-6, IRE1, CHOP, and caspase 12. Densitometric analysis was employed to assess the protein bands. The experiments were conducted independently at least three times, and representative images were presented.

Endoplasmic reticulum (ER) stress refers to a state of cellular stress caused by misfolding and/or excessive accumulation of unfolded proteins within the ER lumen, which can contribute to the progression of kidney disease [23]. As shown in Figure 6 the levels of GRP78, PERK, ATF-6, IRE1, CHOP, and caspase 12 proteins were increased following exposure to cisplatin. Furthermore, GKA4, dead probiotic GKA4, and postbiotic GKA4 decreased the GRP78, PERK, ATF-6, IRE1, CHOP, and caspase 12 protein expression following exposure to cisplatin. The results demonstrate that supplementation with GKA4, dead probiotic GKA4, and postbiotic GKA4 exacerbated kidney damage by suppressing ER-stress-signaling pathways after cisplatin induction (Figure 6).

### 3.8. GKA4, Dead Probiotic GKA4, and Postbiotic GKA4 Alleviate Cisplatin-Induced Renal Transporter Expression

**Figure 7:**
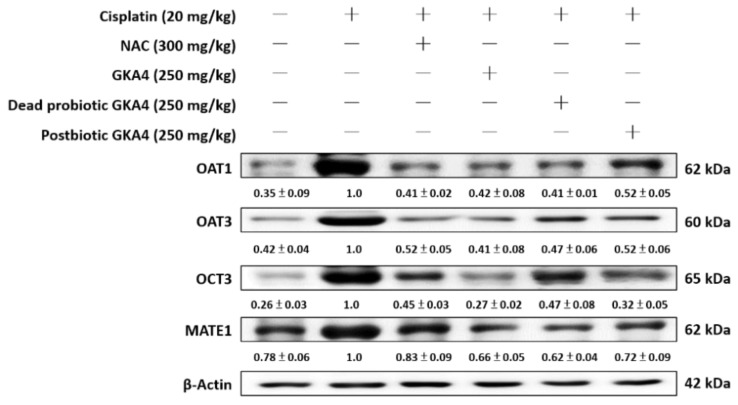
GKA4, dead probiotic GKA4, and postbiotic GKA4 regulated renal transporter expressions following exposure to cisplatin in mice. A Western blot analysis was performed on kidney tissue lysates to evaluate protein expression using antibodies specific to OAT1, OAT3, OCT3, and MATE1. A densitometric analysis was employed to assess the protein bands. The experiments were conducted independently at least three times, and representative images were presented.

Modulated expression of transporters implicated in cisplatin uptake and clearance might act as a mechanism to reduce intracellular accumulation during subsequent exposure [23]. Kidney tissue susceptibility to cisplatin-induced damage hinges significantly on the functionality of renal transporters. The expression levels of these transporters in kidney tissues were examined via Western blotting to elucidate their potential involvement in GKA4, dead probiotic GKA4, and postbiotic GKA4 mechanisms. As shown in Figure 7, the levels of OAT1, OAT3, OCT3, and MATE1 proteins were increased after cisplatin induction. Furthermore, GKA4, dead probiotic GKA4, and postbiotic GKA4 decreased the OAT1, OAT3, OCT3, and MATE1 protein expression following exposure to cisplatin. The results demonstrate that supplementation with GKA4, dead probiotic GKA4, and postbiotic GKA4 increased renal damage by suppressing renal transporter expression after cisplatin induction.

## 4. Discussion

In the human intestine, a multitude of bacterial microorganisms coexist, forming the intestinal bacterial microbiota, which is predominantly comprised of Bacteroidetes, Firmicutes, Proteobacteria, and Actinobacteria. A healthy balance of gut microbiota is essential for sustaining intestinal homeostasis and promoting effective immune function. Dysregulation of gut microbiota is connected to disturbances in the intestinal mucosal barrier and the development of immune-mediated inflammation in several diseases, including kidney disease [23]. AKI symptoms involve a sudden reduction in the glomerular filtration rate (GFR) and an elevation in serum creatinine and BUN levels, culminating in rapid kidney function decline. Chronic kidney disease (CKD) develops when symptoms of AKI persist beyond a duration of three months. The transition from AKI to CKD is closely linked to secondary risk factors like diabetes, hypertension, obesity, and heart disease, either directly or indirectly [28]. Recent research has linked dysbiosis of the intestinal flora with dysfunction in various organs such as the heart, lungs, kidneys, and brain, potentially contributing to the progression of AKI. However, the impact of intestinal dysbiosis and the mechanisms involved in the transition from AKI to CKD remain uncertain or warrant further investigation [28].

Cancer treatment research is advancing rapidly, with chemotherapy drugs continuing to serve as the cornerstone of systemic treatment for numerous cancers. Cisplatin holds a prominent place among chemotherapy agents, being widely employed in the therapeutic arsenal against various cancers affecting both pediatric and adult populations, including ovarian, testicular, bladder, head and neck, breast, and lung malignancies. It features prominently in nearly half of all tumor chemotherapy regimens [29]. However, its application in clinical settings is hampered by various adverse side effects. Presently, approximately 40 adverse reactions associated with cisplatin have been documented. Despite the extensive supportive medical measures provided to cancer patients undergoing cisplatin treatment which enable the utilization of high-dose schedules [30], the adoption of such regimens presents significant challenges, including the occurrence of AKI, diarrhea, neurological complications, and hearing loss. These adverse events often necessitate dose reduction or discontinuation of treatment and have a considerable impact on the patient’s quality of life, potentially exacerbating feelings of depression and anxiety. Currently, there are no effective treatments to prevent these side effects, and existing therapeutic strategies focus on symptom management, offering limited effectiveness. Despite the long-standing clinical use of cisplatin and its well-known anticancer effects, the precise mechanism of cisplatin-induced toxicity remains elusive. There is increasing attention being paid to exploring diverse strategies to uncover the mechanisms of toxicity and address cisplatin-related side effects [31]. While cisplatin mouse models offer a promising avenue for research, significant improvements in treatment outcomes have yet to be realized in clinical practice despite extensive investigations and numerous promising results [32].

Following entry into the cytoplasm, cisplatin becomes activated, with the chlorine atom being replaced by water molecules, resulting in the formation of an electrophilic compound capable of interacting with DNA, thereby inhibiting cell division and prompting apoptosis [27]. Furthermore, the increased solubility of cisplatin facilitates the formation of cisplatin–GSH conjugates, reducing its ability to bind to DNA and providing a shield for dividing cells against cisplatin-induced harm. Consequently, intracellular glutathione levels become depleted. The pathogenesis of cisplatin-induced AKI is multifaceted, involving oxidative stress, inflammation, and apoptosis as key components [28]. Notably, oxidative stress emerges as a central mechanism in AKI. Under physiological conditions, there exists a fine balance between oxidants and antioxidants within the body; nevertheless, perturbation of this equilibrium results in oxidative stress, skewing toward oxidation [29]. Oxidative stress exerts a pivotal influence on AKI by initiating cascades that yield ROS and metabolites serving as ligands for receptor activation. Ample evidence underscores the role of oxidative stress in promoting renal tissue damage through the generation of MDA and the depletion of GSH levels [33]. NAC was employed as a positive control in this study due to being commonly recognized as an antioxidant in clinical trials. Researchers primarily test NAC for its potential in reducing oxidative stress, typically through disulfide bond reduction, scavenging reactive oxygen species, or serving as a precursor for glutathione. Mice treated with NAC in the cisplatin-induced AKI model showed notable improvements in renal function, lessened pathological injury, diminished inflammation, and mitigated oxidative stress [25].

Over the past few years, studies have concentrated on developing more clinically meaningful models of cisplatin-induced renal damage, frequently involving the administration of multiple low-dose treatments over several weeks. These models are capable of recreating CKD progression after AKI, thus improving the likelihood of discovering new therapeutic approaches to cisplatin-induced renal injuries [32]. Despite comprehensive documentation of the condition in animals, our understanding of cisplatin-induced AKI in humans is limited. In clinical practice, GFR and CRE are prioritized over pathological findings in AKI diagnosis [15]. Given the invasiveness of kidney biopsies, they are rarely conducted. Acute tubular necrosis (ATN) is a common AKI indicator, but human nephrotoxic-AKI biopsies show little ATN, unlike animal studies, where ATN is common [34].

Preclinical AKI models are being refined to more accurately reflect patient injuries, ideally matching the clinical scenarios targeted in future trials. The clinical studies that fit these criteria are restricted to four scenarios: AKI from cardiac surgery, contrast agents, cisplatin, and sepsis [35]. The difference between preclinical efficacy studies in animals and clinical AKI trials may stem from several contributing factors. These issues stem from inadequate study designs, the inability to distinguish AKI subtypes, and unreliable biomarkers for predicting outcomes and treatment effects [35]. This analysis revealed that these measures did not predict long-term renal improvements after AKI, pointing to critical gaps in translating preclinical discoveries regarding patient-centered therapeutic approaches. Despite the complexity of the issue, stemming from flaws in both clinical and preclinical designs and methods, we believe that it is a suitable time to reconsider current practices. In the long term, given the complex and multifactorial nature of AKI pathophysiology, a better understanding of the comparative molecular pathogenesis of human and experimental AKI is needed, and preclinical efficacy studies of various AKI models are critical.

The mouse model stands as the predominant animal model for investigating cisplatin-challenged AKI, involving the administration of extremely high doses of cisplatin to mice. The degree of kidney damage is contingent upon the administered cisplatin dose [32]. The study of cisplatin-induced AKI often utilizes two mouse models: the short-term high-dose nephrotoxicity model and the long-term low-dose nephrotoxicity model. For the long-term model, cisplatin is administered at doses ranging from 5 to 15 mg/kg, with treatments occurring two to four times over a 3 to 4-week period. Conversely, short-term models utilize a single high dose of 20–30 mg/kg cisplatin, leading to mortality and nephrotoxicity within 3–7 days post-cisplatin-induced AKI [36]. This study employs a short-term high-dose nephrotoxicity mouse model which has been widely adopted across various research articles. In this model, administration of 20 mg/kg cisplatin induces significant dehydration and impaired activity by the third day along with pronounced clinical symptoms and a notable increase in serum BUN and CRE. In clinical scenarios, patients are typically administered cisplatin at low doses over an extended duration to minimize the risk of nephrotoxicity while maximizing its anticancer effects [36]. In this experiment, mice were treated with GKA4, dead probiotic GKA4, and postbiotic GKA4, which were orally gavaged at a dose of 250 mg/kg per day for 10 consecutive days, starting 7 days before cisplatin injection and ending 3 days after injection to induce AKI.

Our current understanding of the molecular pathways implicated in averting cisplatin-induced AKI remains limited, and effective preventive measures are lacking. The administration of cisplatin has been shown to instigate oxidative stress within kidney tubular cells, subsequently elevating levels of apoptosis and autophagy [37]. Oxidative stress, direct cytotoxicity, and mitochondrial dysfunction may contribute to tubular cell damage. The progression of AKI involves the apoptosis of renal tubular epithelial cells. Thus, addressing tubular injury may be an essential therapeutic focus in the management of cisplatin-induced AKI [29]. Oxidative stress emerges as a key element in cisplatin-challenged AKI. ROS production induced by cisplatin occurs through three mechanisms: interaction with thiol-containing molecules like GSH, disruption of the mitochondrial respiratory chain, and activation of cytochrome P450 (CYP) enzymes leading to ROS generation in microsomes. These processes ultimately result in renal tubular apoptosis [30]. Research indicates that free radicals are produced in large quantities during cisplatin-induced kidney injury, with ROS being a major factor in the resulting acute damage and pathology. Cisplatin-induced oxidative stress has been evaluated using methods like MDA measurement, antioxidant enzyme activity (GPx, catalase, SOD), and GSH levels. Oxidative markers have recently been proposed for non-invasive early detection of cisplatin-induced renal injury. Urinary MDA levels rise within 24 h, suggesting their use as early markers of nephrotoxicity in clinical settings [37]. TBARS is an index of lipid peroxidation, playing a major role in cisplatin-induced nephrotoxicity. MDA serves as a marker for lipid peroxidation and assesses oxidative stress in various tissues. GKA4, dead probiotic GKA4, and postbiotic GKA4 administration resulted in elevated GSH levels and reduced MDA formation following cisplatin induction.

In physiological circumstances, Nrf2, functioning as a transcription factor, is typically sequestered by Keap1 in the cytosol, a binding that is disrupted upon exposure to ROS or electrophiles [38]. After its liberation, Nrf2 migrates to the nucleus and associates with the antioxidant response element (ARE), initiating the transcription of HO-1 and other antioxidant genes. Research findings highlight the importance of Nrf2 as a transcription factor for endogenous antioxidant enzymes, contributing significantly to protection against oxidative and apoptotic injuries [6]. Based on our experimental results, it appears that GKA4, dead probiotic GKA4, and postbiotic GKA4 exert a protective effect by regulating the Nrf2/HO-1 axis and counteracting oxidative stress alterations following cisplatin induction.

A nephrotoxic dose of cisplatin administered in vivo causes a marked increase in kidney apoptosis, with substantial evidence confirming the activation of cisplatin’s intrinsic mitochondrial apoptosis pathway. Renal epithelial cells treated with cisplatin undergo Bax translocation into mitochondria, leading to caspase activation. Moreover, caspase, a fundamental cell death protease, is integral to the execution of apoptosis in cisplatin-induced cell death in renal proximal tubular epithelial cells [39]. Twelve hours after cisplatin is administered to renal epithelial cells, cysteine protease 3 activation is observed. Inhibition of caspase activity has been shown to reduce cell death induced by cisplatin [40]. The study observed cisplatin injections leading to caspase-3 activation and apoptosis, both of which were attenuated by GKA4, dead probiotic GKA4, and postbiotic GKA4. The activation of proapoptotic proteins by cisplatin results in the translocation of cytochrome c into the cytoplasm, subsequently initiating the assembly of a multiprotein complex and the activation of caspases. Hence, the results of our study suggest that GKA4, dead probiotic GKA4, and postbiotic GKA4 alleviate cisplatin-challenged apoptosis by suppressing the caspase-3 pathway.

Autophagy, a process of lysosome-mediated degradation, is essential for recycling cellular components like proteins, lipids, and organelles, contributing significantly to cellular homeostasis [11]. Autophagy is currently acknowledged as a regulated mechanism that greatly affects cell survival in kidney disease. Furthermore, autophagy provides protection against nutrient deficiencies, aging, and pathogen invasion in diseases associated with inflammation [41]. During nutrient insufficiency, autophagy is activated in order to remove defective organelles, produce ATP, and stimulate protein synthesis. However, excessive oxidative stress can cause bodily harm, potentially affecting the expression of autophagy-related proteins such as LC3-II/I, Beclin 1, and p62 [42]. The results indicate that GKA4, dead probiotic GKA4, and postbiotic GKA4 lowered the protein expression of LC3-II, p62, and Beclin 1, suggesting a correlation between autophagy and cisplatin-challenged AKI activation in mice.

AKI typically involves a decline in glomerular filtration and renal excretion, often mandating a decrease in the drug dosage eliminated through renal pathways and/or a prolongation of the dosing frequency. Reductions in renal blood flow, metabolism, and glomerular filtration may contribute to diminished renal filtration and drug elimination, while transporter expression in the kidneys plays a crucial role during renal injury [2]. Proximal tubule cells are critical for the active secretion of xenobiotics and endogenous by-products into the urine, followed by their reabsorption into the body. Secondary active transport systems, including organic anion and cation transporters (OATs, OCTs), facilitate the movement of chemicals across the basolateral membranes into the kidneys. Primary active transporters, namely multidrug resistance-associated proteins (MRP) 2 and 4, multidrug resistance proteins (MDR), breast cancer resistance proteins (BCRPs), and multidrug and toxin extrusion proteins (MATE), enable the efflux of chemicals across the brush border membrane into the urine [16]. Uptake carriers, including organic anion-transporting polypeptides and OAT2, found on the apical membrane, facilitate the reabsorption of chemicals from the filtrate. OAT1 and OAT3 mediate the active transport system, enabling cisplatin to enter kidney epithelial cells effectively. Cisplatin administration resulted in elevated levels of OAT1 and OAT3 protein expression. This facilitates the drug uptake into the tubular cells of the kidney. Because both transporters are involved in cisplatin transport to kidney cells, a reduction in their expression leads to decreased cisplatin uptake [17]. Therefore, interventions aimed at reducing the expression of OATs should be considered a protective measure to shield the kidneys from cisplatin-induced injuries. Furthermore, these membrane proteins transport a range of organic cations with diverse molecular structures. OCT3, found in the small intestine, liver, brain, and other organs, is important for the absorption, excretion, and distribution of cationic drugs [18]. A major transporter for the apical secretion of cisplatin is MATE1. Depletion of MATE1 or the administration of a selective Mate1 inhibitor exacerbates cisplatin-induced nephrotoxicity in mice [19]. Therefore, achieving an equilibrium between uptake and efflux is pivotal in determining the renal accumulation of cisplatin, leading to nephrotoxicity and renal dysfunction.

Lactic acid bacteria (LAB) are widely accepted as beneficial live microorganisms that benefit the host. Recently, in vitro assays like animal models and cell line studies have effectively probed the potential of probiotics, capturing considerable attention in the formulation and advancement of functional foods. The utilization of lactic acid bacteria in food fermentation is widespread, with these microorganisms contributing significantly to flavor profiles and the preservation of fermented items [43]. Generally, these bacteria are acknowledged as being beneficial, with certain strains displaying promise in treating human diseases through the production of heterologous proteins. These proteins consist of lipases, lactase, hormones, interleukins, molecules that stimulate local immune responses, and proteins capable of preventing digestive system diseases [44].

LABs found in the human gut microbiota, including genera such as Lactobacillus, Bifidobacterium, Saccharomyces, Streptococcus, Pediococcus, Leuconostoc, and Bacillus, are classified as probiotics. Notably, *P. acidilactici* displays resistance to stomach acidity [22,25]. Multiple scientific studies have shown that regular consumption of probiotics or their derivatives, particularly lactic acid bacteria, significantly benefits human health by exerting various effects, such as detoxifying xenobiotics and outcompeting pathogenic microbes [24]. LAB strain *P. acidilactici* was administered to laboratory rats, demonstrating its safety for both animal and human consumption. With a higher survival rate in the stomach compared to many other probiotic strains, *P. acidilactici* can more effectively colonize the small intestine and confer gut benefits. *P. acidilactici* is known to produce phenolic compounds with inhibitory effects on molds and fungi in various food products [45]. Furthermore, studies indicate the potential of *P. acidilactici* in managing multiple sclerosis, preventing autoimmune diseases, alleviating constipation, combating diarrhea-inducing enterotoxin *E. coli* and stress, and improving intestinal health. *P. acidilactici* displays exceptional resilience to the acidic environment of the stomach. Its survival rate exceeds that of most other probiotic strains, enabling easier colonization of the small intestine and delivery of gut health benefits. The presence of *P. acidilactici* in the small intestine contributes to improving health by producing lactic acid and a diverse range of antimicrobial compounds which combat harmful pathogens in the gut [46]. Numerous studies on *P. acidilactici* are currently underway, with accumulating evidence suggesting its capacity to confer a diverse array of beneficial effects.

Metabolites and by-products, termed postbiotics, are generated and released by food-grade microorganisms, notably LAB strains, during their growth in culture or food matrices and in the gastrointestinal tract [47]. Postbiotics include soluble factors that are produced by viable bacteria or released upon bacterial lysis, such as cell wall fragments, exopolysaccharides, enzymes, short-chain fatty acids, and bacterial lysates. The principal components of postbiotics are organic acids, bacteriocins, bacteriocin-like inhibitory substances (BLISs), and other compounds like reuterin, reuterocyclin, diacetyl, and alcohols [48]. Despite the extensive research on certain metabolites in postbiotics, such as bacteriocins or BLISs, there is still a need for thorough characterization, identification, and quantification of these components. In addition, heat-inactivated bacteria possessing health-promoting properties are also encompassed within the category of postbiotics. Postbiotics are increasingly being integrated into functional foods, reflecting a growing trend in the food industry. Numerous studies have highlighted the multifaceted functional properties of postbiotic molecules, which encompass antibacterial, antioxidant, anti-obesity, immunomodulatory, anticancer, and antiallergic effects. However, further research is needed to fully elucidate the efficacy of postbiotics in food formulations [49].

The overabundance of free radicals can lead to tissue damage, primarily via the generation of hydroxyl radicals and other oxidants. This damage induced by free radicals is considered to be a significant factor in the pathogenesis of many diseases [4]. Probiotics have been employed therapeutically to modulate immunity, enhance digestive processes, reduce cholesterol, manage rheumatoid arthritis, and prevent cancer. The antioxidative prowess of LAB is linked to their capacity to produce antioxidant enzymes and GSH, which govern host antioxidant activity and augment antioxidant metabolite concentrations throughout the body [9,26]. This influences the host by modulating signaling pathways, inhibiting enzymes that produce ROS, and regulating the composition of gut microbiota. The chemopreventive efficacy of LAB has been validated in various in vitro, in vivo, and clinical human studies, acting through mechanisms such as the modulation of gastrointestinal microbiota, enhancement of immune responses, and antioxidant and antiproliferative effects [50]. LAB have been found to possess antioxidant capabilities, as per the research, owing to their ability to chelate metals and scavenge ROS, thus playing a critical role in disorders correlated with disruptions in the intestinal microbiota, such as inflammatory conditions, diabetes, and cancer [51]. Evidence from clinical research suggests that probiotics, prebiotics, and synbiotics are beneficial for improving the metabolic profile of individuals with CKD. This includes a reduction in inflammatory markers such as *C*-reactive protein (CRP); oxidative stress indicators like MDA, GSH, and total antioxidant capacity; and enhancement of lipid profiles comprising total cholesterol, triglycerides, low-density lipoprotein cholesterol, and high-density lipoprotein cholesterol.

## 5. Conclusions

In this article, we illustrate how GKA4, dead probiotic GKA4, and postbiotic GKA4 regulate oxidative stress following cisplatin-induced AKI by mitigating kidney histopathologic alterations. These substances demonstrate robust antioxidant effects against cisplatin-related AKI that are accomplished through the inhibition of apoptosis, autophagy, ER stress, and the dysfunction of renal transporters. The present investigation indicates that GKA4, dead probiotic GKA4, and postbiotic GKA4 confer renoprotection only in a murine model of cisplatin-mediated nephrotoxicity. Additional studies are necessary in order to confirm their efficacy. In summary, GKA4, dead probiotic GKA4, and postbiotic GKA4 administration attenuates oxidative stress in cisplatin-induced AKI, thus preventing renal injury.

## Figures and Tables

**Table 1:** The oral delivery of GKA4, dead probiotic GKA4, and postbiotic GKA4 treatment results in variations in body weight and the renal index during cisplatin-induced nephrotoxicity. Means ± S.E.M (*n* = 5) are shown in the presentation of the data. Statistical significance (*p* < 0.001) is indicated by ^###^ when compared with the control group sample. *** *p* < 0.001 compared with the cisplatin group. To determine the kidney index, the kidney weight is divided by the body weight.

Groups	Initial Body Weight (g)	Final Body Weight (g)	Kidney Index (mg/g)
Control	36.25 ± 0.67	39.72 ± 1.59	1.30 ± 0.07
Cisplatin (20 mg/kg)	35.90 ± 0.82	34.45 ± 1.06	1.76 ± 0.08 ^###^
Cisplatin (20 mg/kg) + NAC (300 mg/kg)	36.12 ± 1.87	36.90 ± 0.40	1.44 ± 0.06 ***
Cisplatin (20 mg/kg) + GKA4 (250 mg/kg)	35.82 ± 0.85	36.43 ± 0.78	1.47 ± 0.06 ***
Cisplatin (20 mg/kg) + Dead probiotic GKA4 (250 mg/kg)	35.88 ± 1.94	36.60 ± 0.63	1.42 ± 0.05 ***
Cisplatin (20 mg/kg) + Postbiotic GKA4 (250 mg/kg)	35.63 ± 0.59	36.40 ± 0.68	1.46 ± 0.06 ***

## Data Availability

The original contributions presented in the study are included in the article, further inquiries can be directed to the corresponding author.
